# Editorial: The influence of SARS-CoV-2 infection and long-COVID on the incidence of viral coinfection

**DOI:** 10.3389/fimmu.2026.1937175

**Published:** 2026-08-28

**Authors:** Roberta Rizzo, Rebecca Jane Cox, Alexis Hipólito García, Juan Bautista De Sanctis

**Affiliations:** 1 Department of Environmental and Prevention Sciences, University of Ferrara, Ferrara, Italy; 2 Influenza Center, Department of Clinical Science, University of Bergen, Bergen, Norway; 3 Institute of Immunology “Dr Nicolás Enrique Bianco C”, Faculty of Medicine, Universidad Central de Venezuela, Caracas, Venezuela; 4 Institute of Molecular and Translational Medicine, Faculty of Medicine and Dentistry, Palacky University, Olomouc, Czechia; 5 Czech Advanced Technology and Research Institute, Palacký University in Olomouc, Olomouc, Czechia

**Keywords:** co-infection, cytokines, immune response, machine learning, RNAseq, SARS-CoV-2, T cell receptor (TCR)

## Introduction

The SARS-CoV-2 virus has been in circulation since 2019, leading to over 700 million documented cases worldwide, with a mortality rate estimated at approximately 1%, according to the World Health Organization (1). In individuals in good health, the infection generally elicits a transient, relatively modest cellular and humoral immune response against the virus (2). It is estimated that 10-20% of individuals previously infected with SARS-CoV-2 will develop long COVID-19 (3, 4). However, the underlying causes of this condition remain poorly understood. In addition, patients with COVID-19 and long COVID-19 may be more susceptible to other infections (5). Potential explanations may include the persistence of SARS-CoV-2 within the organism, the emergence of variants with an abundance of spike mutations, and an impaired immune response following viral infection.

The aim of the topic was to conduct a more detailed analysis of SARS-CoV-2 infection, the risk of developing long COVID, and potential susceptibilities to other infections. In this topic, we had 20 contributions, published across different areas, including omics, co-infection, and machine learning.

Zhou et al. analyzed scRNA-seq, bulk RNA sequencing, and proteomics data to identify key COVID-19 biomarkers. They found that BTD, CFL1, PIGR, and SERPINA3 could serve as potential auxiliary diagnostic markers related to CD8+ T cells. In addition, Mercer et al. conducted a miR-proteome analysis to explore how miR regulation relates to COVID-19 severity. They found negative correlations between miRs and proteins, suggesting that miRs regulate pathways linked to severity. This reveals insights into immune response regulation and potential therapeutic targets for COVID-19.

The effects of cytokines on immune cell populations in COVID-19 patients were assessed by (Choto et al.). The authors analyzed levels of IFN-γ, IL-6, IL-10, IL-17, and TNF-α. They found that IL-6 levels and peripheral blood leukocyte counts, including platelet counts, were critical. Additionally, the quantity of immature granulocytes was associated with the groups defined by IL-10 levels. Abdullah et al. studied the impact of COVID-19 on cytokines and gene polymorphisms in patients with type 1 diabetes. Machine learning analyses identified IL-18, CRP, and IL-6 as key biomarkers of COVID-19 severity, with IL-18 being the most important.

In long COVID patients, Fricke et al. found downregulation of pro-inflammatory genes in classical monocytes, including IL1β and CXCL2. Their analysis suggested reduced inflammasome activity (via SNAI1) and impaired monocyte differentiation (via ATF2), leading them to conclude that circulating monocytes are either immature or exhausted.

Markey et al. analyzed T cell and TCR data from hospitalized Omicron patients and found that a significant proportion of TCRs targeted epitopes across the SARS-CoV-2 genome, including non-structural proteins implicated in viral replication and immune evasion. Their findings highlight how T cell protective immunity is influenced by both the viral replication cycle and HLA type, affecting TCR affinity and clonotype formation. Furthermore, Wen, Y (6). et al. evaluated the heterogeneity and transcriptomic characteristics of Tregs in COVID-19 patients and concluded that Tregs may be activated through the bystander effect, as evidenced by the analysis of TCR clonotype characteristics. The analysis proposed possible analyses of treatment strategies in SARS-CoV-2-infected patients.

The response against SARS-CoV-2 infection in the elderly population after 6 months was analyzed by (Luo et al.). The decline in memory B cells and CD69+ and CD38+ T cells, along with the rise in PD-1+ and CTLA-4+ in CD4+/CD8+ T cells and double-negative B cells, suggests that immune responses to BA.5 infection may diminish more quickly in the elderly. Luo et al. developed an explainable machine learning-based mortality risk stratification for older adults with COVID-19, pinpointing core immunological biomarkers and revealing dose-threshold effects. The model identified 10 key features: basophil percentage, C-reactive protein, procalcitonin, D-dimer AST/ALT ratio, cardiac troponin I, standard bicarbonate, age, aspartate transaminase, and oxygen saturation. Those parameters were considered critical for calculating patient survival.

Chen et al. reviewed mitochondrial metabolism in COVID-19, highlighting the need for long-term patient data and addressing analytical limitations. They suggested that nutrients like CoQ10, NAC, and creatine could help restore energy metabolism and reduce oxidative stress. The authors emphasized the importance of interdisciplinary collaboration for multi-omics studies of mitochondrial-epigenetic interactions, targeted antioxidants, and optimized nutrient delivery systems.

Marandu et al. found that vaccination benefited patient recovery from viral infections. While CD4 cells were impacted only during active infection, changes in B and NK cells were noted in the vaccinated group. Additionally, T cell hyperresponsiveness was seen in patients with severe disease.

Fialho et al. studied the role of neutralizing antibodies against COVID-19 and its variants. They concluded that while COVID-19 vaccination effectively generates protective immunity, challenges remain due to immune evasion by Omicron subvariants and waning immunity. These findings highlight the need for updated booster strategies and sustained public health efforts for full vaccine coverage and long-term protection.

Yin et al. analyzed data from large-scale serosurveys and found that median IgG antibody levels declined after 3 months. Higher levels were linked to prior infection and vaccination, while older age was associated with annual decay, especially in adults ≥60 years. The IgG threshold for protection against XBB reinfection was 400 AU/mL, with each additional vaccination reducing reinfection risk by 13–15%.

Bansai A (7) emphasized that no single protein can diagnose post-COVID patients; phenotype-linked multi-analyte panels show the most promise. Effective post-COVID research requires standardized case definitions, cross-platform validation, and the integration of coagulation assays and extracellular vesicle profiling to improve tissue signal detection.

## Co-infection

Cruz-Altamirano et al. conducted a pilot proteomic analysis on immune dysregulation in dengue following prior SARS-CoV-2 infection. They concluded that previous SARS-CoV-2 exposure may increase dengue severity through cross-reactive antibodies, shifting the immune response toward Th2 and enhancing viral replication. Furthermore, Parra-González et al. and colleagues reviewed the interactions between dengue and SARS-CoV-2 and their impact on clinical outcomes. They found that co-infection may worsen inflammation, increase vascular and organ damage, and complicate treatment. However, definitive evidence for antibody-dependent enhancement (ADE) remains inconclusive, highlighting the need for further mechanistic research.

The proteomic profile analysis of hospitalized HIV- and SARS-CoV-2 coinfected patients was studied by (Zhang et al.). The proteomic profile highlighted disruptions in immunomodulation, thrombosis, and viral entry pathways in people living with HIV caused by COVID-19. However, a larger trial is needed to ascertain the effect of coinfection on immune response.

The assessment of Programmed cell death markers in COVID-19 survivors with and without sepsis was analyzed by Mallapu G et al. (8). The analysis revealed two molecular signatures linked to disease severity. The first signature, showing high levels of secreted programmed cell death markers (IL-1-β, IFN-γ, MHC I-A, MHC II-DRB1), was found in both COVID-19-Sepsis and no Sepsis cohorts. The second signature, containing cellular programmed cell death markers (caspase-1, caspase-3, MLKL, p62/SQSTM1), was specific to the COVID-19-Sepsis cohort. These signatures may serve as biomarkers for disease severity and guide therapeutic interventions in critical care.

The relationship between SARS-CoV-2 infection and Streptococcus pneumoniae colonization and disease severity was analyzed by (Calvo–Silveira et al.). Patients with co-infections have higher co-morbidities and severe clinical presentations. Interestingly, non-pharmacological measures may disrupt the S. pneumoniae dynamics, and COVID vaccination can prevent the possibility of coinfection and decrease disease severity.

Currey et al. studied post-viral sequelae using a mouse model of influenza and SARS-CoV-2 in C57BL/6 mice. They infected the mice with sublethal doses of SARS-CoV-2 (MA30) or influenza A (PR8) and analyzed lung and brain tissues at 14-, 21-, and 28-days post-infection using histological analysis and bulk-RNA sequencing. They were able to establish distinct tissue-specific trajectories of long-term pathology following SARS-CoV-2 and influenza infection and provide a foundation for dissecting the mechanisms of post-viral lung and brain disease.

The topic presents a compelling collection of manuscripts that cover several critical aspects of SARS-CoV-2 infection, vaccine evaluation, and coinfection, thereby enabling readers to gain a comprehensive understanding of this intricate phenomenon. Notably, there are various proposals for conducting rational risk analyses regarding disease severity, evaluating the immune response to the virus, and assessing the effects of vaccination. Furthermore, suggestions for therapeutic targets that may prove beneficial for future investigations, including those related to long COVID, have been identified. Despite the pandemic originating over six years ago, several unresolved issues persist, particularly regarding post-infection consequences. Consequently, further research is essential to unravel the complex elements associated with infection and coinfection.

## References

[1] Available online at: https://data.who.int/dashboards/covid19/cases (Accessed July 8, 2026).

[2] De Sanctis JB García AH Moreno D Hajduch M . Coronavirus infection: an immunologists' perspective. Scand J Immunol. (2021) 93:e13043. doi: 10.1111/sji.13043 33783027 PMC8250184

[3] Fleischer NL Slocum E Patel A Xie Y McKane P Lyon-Callo S . Long COVID and new onset disability nearly 2 years after initial infection. Am J Prev Med. (2025) 68:1168–72. doi: 10.1016/j.amepre.2025.02.013 40054707

[4] Fischer A Zhang L Elbéji A Wilmes P Snoeck CJ Larché J . Trajectories of persisting covid-19 symptoms up to 24 months after acute infection: findings from the Predi-Covid cohort study. BMC Infect Dis. (2025) 25:603. doi: 10.1186/s12879-025-11023-0 40281467 PMC12023393

[5] Carazo S Ouakki M Nicolakakis N Falcone EL Skowronski DM Durand MJ . Long COVID risk and severity after COVID-19 infections and reinfections: a retrospective cohort study among healthcare workers. Int J Infect Dis. (2025) 159:108012. doi: 10.1016/j.ijid.2025.108012 40783164

[6] Wen Y Zhao J Zhang Z . Heterogeneity and longitudinal transcriptomic characteristics of Tregs in COVID-19 patients. Front Immunol. (2025) 16:1548173. doi: 10.3389/fimmu.2025.1548173 40114921 PMC11922936

[7] Fialho MJ Silva CJF Miranda GBA Assis ISS Moreno AM Moura PH . The landscape of neutralizing antibodies against SARS-CoV-2 variants: insights from a stratified vaccination cohort. Front Immunol. (2025) 16:1667392. doi: 10.3389/fimmu.2025.1667392 41080532 PMC12511102

[8] Bansal A . Proteomic landscapes of post-COVID condition: biomarkers and translational pathways. Front Immunol. (2026) 17:1783192. doi: 10.3389/fimmu.2026.1783192 42421959 PMC13342817

